# Examining inequalities in spatial access to national health insurance fund contracted facilities in Kenya

**DOI:** 10.1186/s12939-024-02171-x

**Published:** 2024-04-18

**Authors:** Jacob Kazungu, Angela K. Moturi, Samson Kuhora, Julia Ouko, Matthew Quaife, Justice Nonvignon, Edwine Barasa

**Affiliations:** 1 Health Economics Research Unit, KEMRI Wellcome Trust Research Programme, Nairobi, Kenya; 2 Population & Health Surveillance Group, KEMRI Wellcome Trust Research Programme, Nairobi, Kenya; 3 National Health Insurance Fund, Nairobi, Kenya; 4 Department of Epidemiology and Population Health, London School of Hygiene and Tropical Medicine, London, UK; 5 Department of Health Policy, Planning and Management, School of Public Health, University of Ghana, Legon, Accra Ghana; 6 Health Economics and Financing Programme, Africa Centres for Disease Control and Prevention, Addis Ababa, Ethiopia; 7 Center for Tropical Medicine and Global Health, Nuffield Department of Medicine, University of Oxford, Oxford, UK

**Keywords:** Spatial access, Inequalities, Health facility, National Health Insurance Fund, Travel time, Universal health coverage, Kenya

## Abstract

**Background:**

Kenya aims to achieve universal health coverage (UHC) by 2030 and has selected the National Health Insurance Fund (NHIF) as the ‘vehicle’ to drive the UHC agenda. While there is some progress in moving the country towards UHC, the availability and accessibility to NHIF-contracted facilities may be a barrier to equitable access to care. We estimated the spatial access to NHIF-contracted facilities in Kenya to provide information to advance the UHC agenda in Kenya.

**Methods:**

We merged NHIF-contracted facility data to the geocoded inventory of health facilities in Kenya to assign facility geospatial locations. We combined this database with covariates data including road network, elevation, land use, and travel barriers. We estimated the proportion of the population living within 60- and 120-minute travel time to an NHIF-contracted facility at a 1-x1-kilometer spatial resolution nationally and at county levels using the WHO AccessMod tool.

**Results:**

We included a total of 3,858 NHIF-contracted facilities. Nationally, 81.4% and 89.6% of the population lived within 60- and 120-minute travel time to an NHIF-contracted facility respectively. At the county level, the proportion of the population living within 1-hour of travel time to an NHIF-contracted facility ranged from as low as 28.1% in Wajir county to 100% in Nyamira and Kisii counties. Overall, only four counties (Kiambu, Kisii, Nairobi and Nyamira) had met the target of having 100% of their population living within 1-hour (60 min) travel time to an NHIF-contracted facility. On average, it takes 209, 210 and 216 min to travel to an NHIF-contracted facility, outpatient and inpatient facilities respectively. At the county level, travel time to an NHIF-contracted facility ranged from 10 min in Vihiga County to 333 min in Garissa.

**Conclusion:**

Our study offers evidence of the spatial access estimates to NHIF-contracted facilities in Kenya that can inform contracting decisions by the social health insurer, especially focussing on marginalised counties where more facilities need to be contracted. Besides, this evidence will be crucial as the country gears towards accelerating progress towards achieving UHC using social health insurance as the strategy to drive the UHC agenda in Kenya.

## Background

Since the adoption of the Sustainable Development Goals (SDGs), there is a growing consensus for countries to make reforms towards meeting all 17 goals. In the health sector, countries have committed and are increasingly making progress towards attaining Universal Health Coverage (UHC) [1–3]. UHC calls on countries to reform their health systems to ensure that their population can access good quality preventative, promotive, curative, and rehabilitative services whenever they need them without exposing them to financial hardship [4]. While the quality of care and financial risk protection are fundamental UHC goals, ensuring that the population has access to healthcare services is also crucial [5].

Penchansky and Thomas have defined access to healthcare to reflect five dimensions related to the availability, accessibility, affordability, acceptability and accommodation of care [6]. While all dimensions are crucial to ensuring that the population gains access to care, often, the availability and accessibility dimensions (hereafter referred to as spatial accessibility) are overlooked [7]. Guagliardo et al. defined availability as the number of local services from which a client can choose [7]. Spatial accessibility refers to the difficulty or ease (travel impedance) of a user moving from where they need healthcare services (such as their household) to the location where the healthcare services are provided (service provider location) [7]. Both availability and spatial accessibility are essential components of UHC as they directly influence both the level of financial risk protection and health outcomes. For instance, on financial risk protection, a study that examined the impoverishing effects of catastrophic healthcare payments in Kenya found that transport costs to and from health facilities accounted for 1.46% of the incidence of catastrophic health expenditures with the incidence being over five times in the rural settings (2.02%) compared to urban areas (0.38%) [8]. In another study, Oyando et al. reported that transport costs alone accounted for 22.6% of the total direct costs for diabetes [9]. On health outcomes, spatial accessibility has been shown to influence child and neonatal mortality [10–12], maternal and newborn health [13, 14] and childhood vaccination [15–17].

In Kenya, several studies have estimated spatial accessibility to health facilities [15, 18–20]. The most recent study by Moturi et al. reported that the national average travel time to the nearest health facility was 130 min to public facilities, 254 min to private facilities, and 128 min to both public and private facilities [19]. Additionally, 89.4, 80.5 and 89.6% of the population were found to be living within the recommended 1-hour travel time to a public, private and both public and private facility, respectively. While such evidence is important for decision-making, it does not fully provide information to advance the UHC agenda in Kenya given that (1) not all health facilities in Kenya are contracted by the National Health Insurance Fund (NHIF) thus placing availability and accessibility barriers to accessing care, and (2) the NHIF has been selected as the vehicle to drive the UHC agenda in Kenya [21] and has key decisions around identifying, selecting, and contracting healthcare providers [22].

The NHIF is a social health insurer in Kenya that was established in 1966 [22, 23]. Over the years, the NHIF has undergone several reforms [24] aiming at transforming it into a strategic purchaser to streamline its decisions on the design of health benefits package, decisions of the set of health providers to buy from and the modalities for reimbursing healthcare providers (contracting and provider payment mechanisms) [25]. Consequently, the NHIF contracts healthcare providers either under one of the two contract categories: comprehensive or non-comprehensive contracts [26]. Comprehensive contracts are mostly with public facilities and some select low-cost private and faith-based facilities that do not permit co-payment for inpatient care. On the other hand, non-comprehensive contracts include some level of co-payment where NHIF covers the cost of specified services (such as bed charges), and the member tops up the rest of the charges, especially in high-end private or faith-based facilities [26].

Kenya has a pluralistic health system characterised by a mix of public, private and faith-based providers organised into four tiers of care comprised of six levels [27]. Tier 1 comprises community health units (Level 1); Tier 2 comprises primary care facilities made up of Level 2 (Dispensaries and clinics) and levels 3 facilities (health centres); Tier 3 comprises secondary care facilities made up of Level 4 (sub-county hospitals) and Level 5 (county referral) facilities; and Tier 4 is made up of Level 6 facilities which make up the national referral facilities in Kenya. As of 16th October 2023, there were a total of 14,403 health facilities in Kenya with the public sector accounting for 46.0% (6,623 facilities), private-for-profit accounting for 46.9% (6,764 facilities) and faith-based facilities accounting for 7.1% (1,016) facilities [28]. The NHIF is the largest health insurer covering 24% of Kenyans [29].

Given the role of NHIF in identifying, selecting and contracting health facilities in Kenya, it is imperative to understand the extent to which the distribution of NHIF-contracted facilities may promote or undermine spatial access primarily to inform decisions about whom to buy from as a move to (1) remove access barriers, (2) meet the government target to have 100% of the population living within 1 h travel time (5 km) to a health facility, and (3) to enhance equity in the distribution of contracted facilities across both poor and better-off populations [30].

Against this backdrop, this study examined the inequalities in the spatial access to NHIF-contracted facilities in Kenya. We computed the proportion of the population living within a 1- and 2-hour travel time to an NHIF-contracted facility nationally and at the county level.

## Methods

### Data assembly

#### Health facilities

We downloaded a list of all facilities in Kenya from the Kenya Master Health Facility List (KMHFL) database (http://kmhfl.health.go.ke/#/facility_filter/results) [28]. The KMHFL is a database that, in theory, contains a list of all health facilities and community units in Kenya with descriptions of their name, location, types of services offered, ownership, administrative location (County, constituency, sub-county, and ward) and a unique master facility list (MFL) code. The list contained 14,403 facilities as of 16th October 2023.

We then obtained data on the facilities contracted by NHIF to provide outpatient and inpatient services as well as the contract types: comprehensive or non-comprehensive through a data request to the NHIF. The inventory contains facility names in addition to the relevant administrative information. This list is updated periodically as new facilities are contracted or others deregistered. Consequently, the data used in this analysis was as of December 2022. Initially, the list contained a total of 7,570 facilities. This list was then matched to the KMHFL to assign relevant information on ownership, and levels and also update or confirm the administrative location attributes.

The most rudimentary analysis of spatial accessibility requires an understanding of the exact location of facilities, which are not available in either the NHIF or the KHMFL. Thus, facility coordinates were obtained from a recently developed master health facility list for Kenya assembled in 2021 [19]. This inventory collated and merged the two main listings managed by the Ministry of Health including the KMHFL and a listing of operational facilities from Kenya’s routine health information system reporting platform. This database included both private and public sector providers with health facility details including, MFL code, ownership, Kenya Essential Package for Health (KEPH) level and geographic coordinates, confirmed using digital gazetteers and GPS coordinates sourced from previous mapping exercises [20]. The NHIF facilities were then linked to this database using the name and unique identifier (MFL code). Methods used in mapping these facilities were employed to map NHIF facilities that may not be captured in the 2021 database. We excluded some facilities that were NHIF-contracted from the initial list of 7,570 facilities. Excluded facilities included duplicates (429), prisons and Kenya Defence Forces Memorial facilities (31) and specialised facilities such as those offering dental, diagnostics and cancer specialised services (2,154). These were excluded either because (1) they are not accessible to the general public, (2) they were not geocoded (1,098), and (3) they do not offer general outpatient or inpatient services for the general population. Overall, we included a total of 3,858 NHIF-contracted facilities in this analysis.

#### Population data

The 2022 population data for Kenya were obtained from the WorldPop database. The rationale for using this resource is that population data is presented at 100 m spatial resolution, allowing for an understanding of heterogeneous population distribution in a manner not possible with aggregate census count data. Details for its construction are presented elsewhere [31, 32], but in brief, subnational level data are disaggregated to 100 m square grids using a random forest algorithm that used the likelihood of finding people at different locations based on factors such as land use, elevation, and nighttime lights.

#### Covariates data

We included three major covariates that have been shown to influence spatial access [33], namely: (1) road network, (2) elevation, and (3) land cover. We assembled data from various sources to incorporate these three key covariates. For the road network, a database of roads including their attributes previously assembled at the Population Health Unit (PHU) at KEMRI Wellcome Trust Research Programme was used for this analysis [18]. For elevation, slope data based on a Digital Elevation Model at 3om spatial resolution were obtained from the Regional Center for Mapping of Resources for Development (RCMRD) data Geoportal (http://geoportal.rcmrd.org/) which provides open access spatial data. On land cover, land use data developed by the European Space Agency Sentinel-2 imagery was sourced for the year 2021 at 10 m spatial resolution (https://worldcover2021.esa.int/viewer) [34]. The data details the main land cover classes including water, trees, grass, flooded vegetation, crops, shrubs, built-up area, and bare ground which influence travel in places without a road network. Lastly, a previously assembled database of protected areas and water bodies that have been used in similar analyses to simulate physical barriers to access [19, 35–37] was used in the current work. The effect of seasonality and traffic jams was not accounted for in this analysis due to data limitations.

### Analysis

#### Spatial access

A cost-distance algorithm that models a composite of walking and motorised time to the nearest NHIF-contracted outpatient facility was used as a measure of geographical access. This model developed using the WHO AccessMod tool version 5.8 [38] takes into account the elevation, land cover, and proximity to roads while treating the transport barriers such as lakes and protected areas impassable unless a bridge or road is crossing the barrier that enables access. These landscape features were used to create a cost friction surface, which defines the difficulty in moving through each 1 km square grid, as defined by the speeds. Speeds across different roads and land use were obtained from recent studies [18, 39, 40]. The cost friction surface and location of NHIF-contracted outpatient providers were then used to estimate the time in hours/minutes needed to travel to the nearest NHIF-contracted facility. We estimated the proportion of the population living within 60- and 120-minute travel time to an NHIF-contracted facility, comprehensive NHIF-contracted facility, outpatient NHIF-contracted facility and inpatient NHIF-contracted facility. We also estimated the average travel time to (1) any NHIF-contracted facility, (2) NHIF-contracted outpatient facility and (3) NHIF-contracted inpatient facility. Good access was defined as living within 1-hour (60-minutes) travel time to the nearest NHIF-contracted facility as recommended in the Kenya Health Sector Strategic Plan [41]. We also derived county-level proportions of the population within this travel time threshold to define the accessibility quotients to NHIF-contracted facilities at a sub-national level to inform planning.

## Results

### Distribution of NHIF-contracted facilities in Kenya

**Fig. 1:**
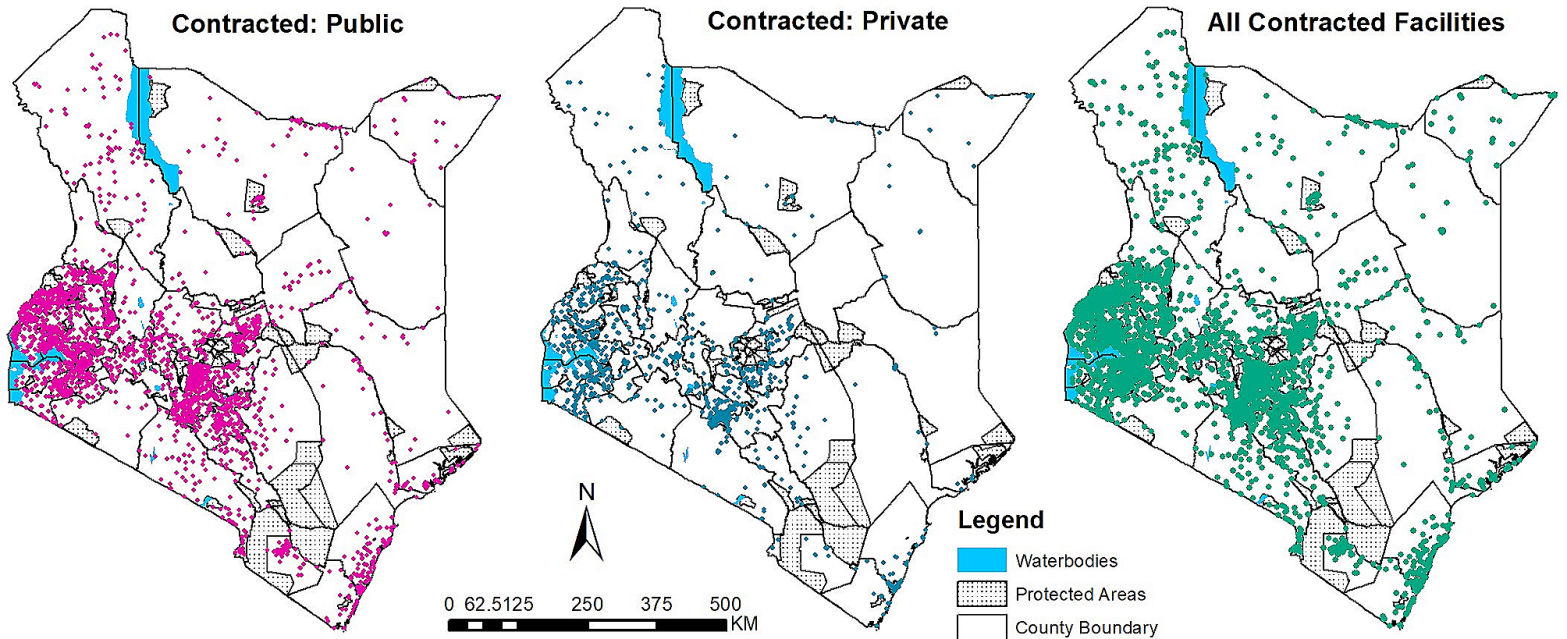
NHIF-contracted health facilities spatial distribution in 2022 by sector: Public (*n* = 2,253), Private^a^ (*n* = 1,605), and Total (*n* = 3,858). ^a^– Contains both Private-for-profit (*n* = 1,295) and Faith-based (*n* = 310)

We included a total of 3,858 NHIF-contracted facilities in this analysis. Out of these, 58.5% (2,253) were public facilities, 8.0% (310) were faith-based facilities (FBO) and 33.5% (1,295) were private facilities (Table 1; Fig. 1). Besides, 99.5% (3,838) of the facilities were contracted for outpatient services while 84.7% were contracted for inpatient services. Table 1 summarises the number of NHIF contracted facilities by contract type, level of care, and ownership.

**Table 1:** Distribution of NHIF contracted facilities by contract type, outpatient, and inpatient care facilities

Level of care	Ownership	Contract type - Comprehensive		Contract type - non-Comprehensive		Outpatient		Inpatient	
		Number	Percentage (%)	Number	Percentage (%)	Number	Percentage (%)	Number	Percentage (%)
Level 2	Public	1,391	65.9	0	0.0	1,391	64.6	1,327	67.4
	Private	596	28.2	45	91.8	634	29.5	531	27.0
	FBO	123	5.8	4	8.2	127	5.9	110	5.6
Level 3	Public	546	52.0	2	8.7	548	51.5	507	56.6
	Private	404	38.5	20	87.0	416	39.1	326	36.4
	FBO	100	9.5	1	4.3	100	9.4	63	7.0
Level 4	Public	302	54.7	0	0.0	300	49.6	206	52.8
	Private	177	32.1	50	89.3	227	37.5	136	34.9
	FBO	73	13.2	6	10.7	78	12.9	48	12.3
Level 5	Public	8	66.7	0	0.0	8	57.2	6	66.7
	Private	2	16.7	1	50.0	3	21.4	1	11.1
	FBO	2	16.7	1	50.0	3	21.4	2	22.2
Level 6	Public	4	100.0	0	0.0	3	100.0	3	100.0
**Total**	Public	2,251	60.4	2	1.5	2,250	58.6	2,049	62.7
	Private	1,179	31.6	116	89.2	1,280	33.4	994	30.5
	FBO	298	8.0	12	9.3	308	8.0	223	6.8
**Grand-total**		**3,728**		**130**		**3,838**		**3,266**	

### Spatial access to NHIF-contracted facilities nationally and at the county level in Kenya

**Fig. 2:**
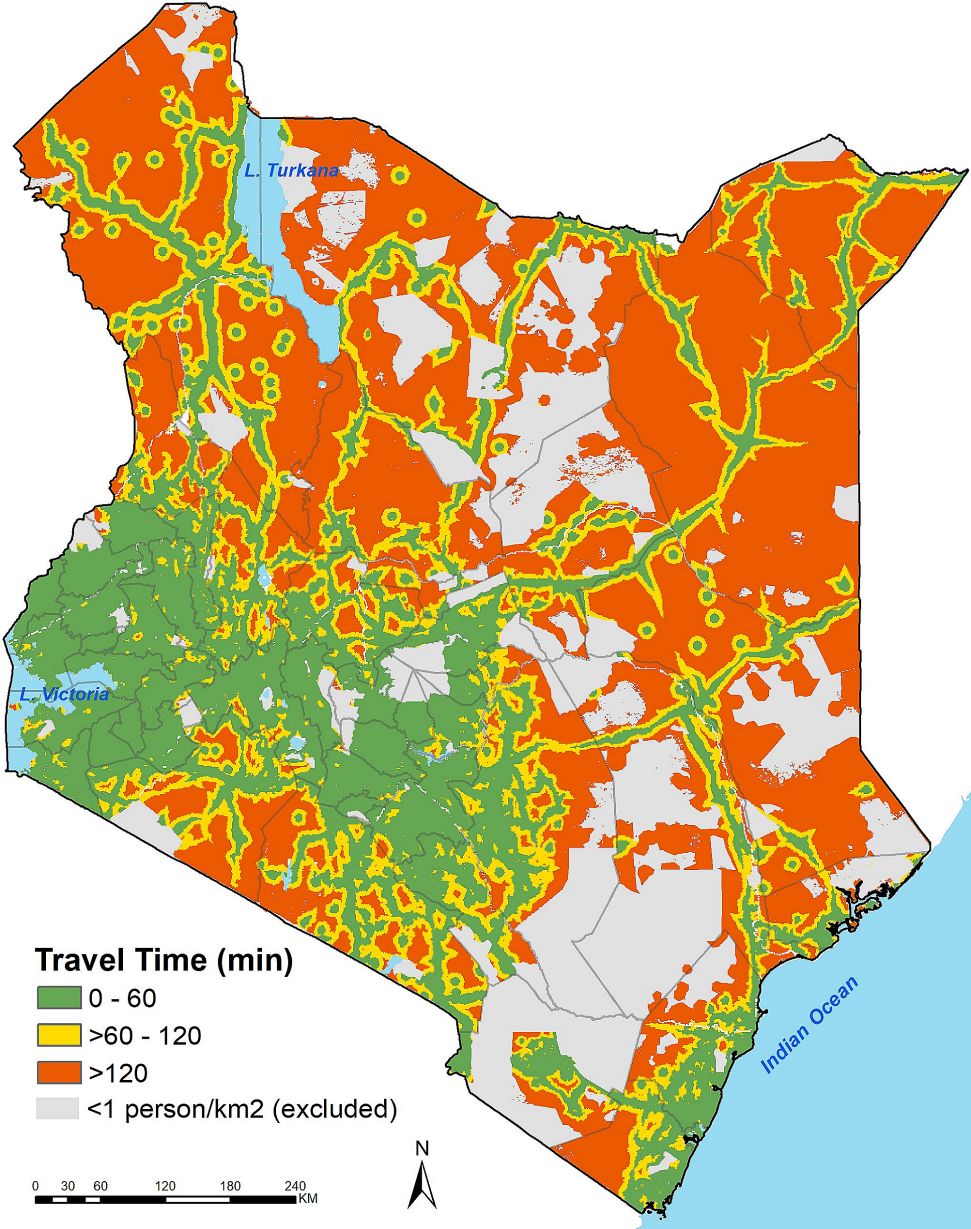
Travel time to the nearest NHIF-contracted facility binned based on travel time and population density in Kenya in 2022

**Fig. 3:**
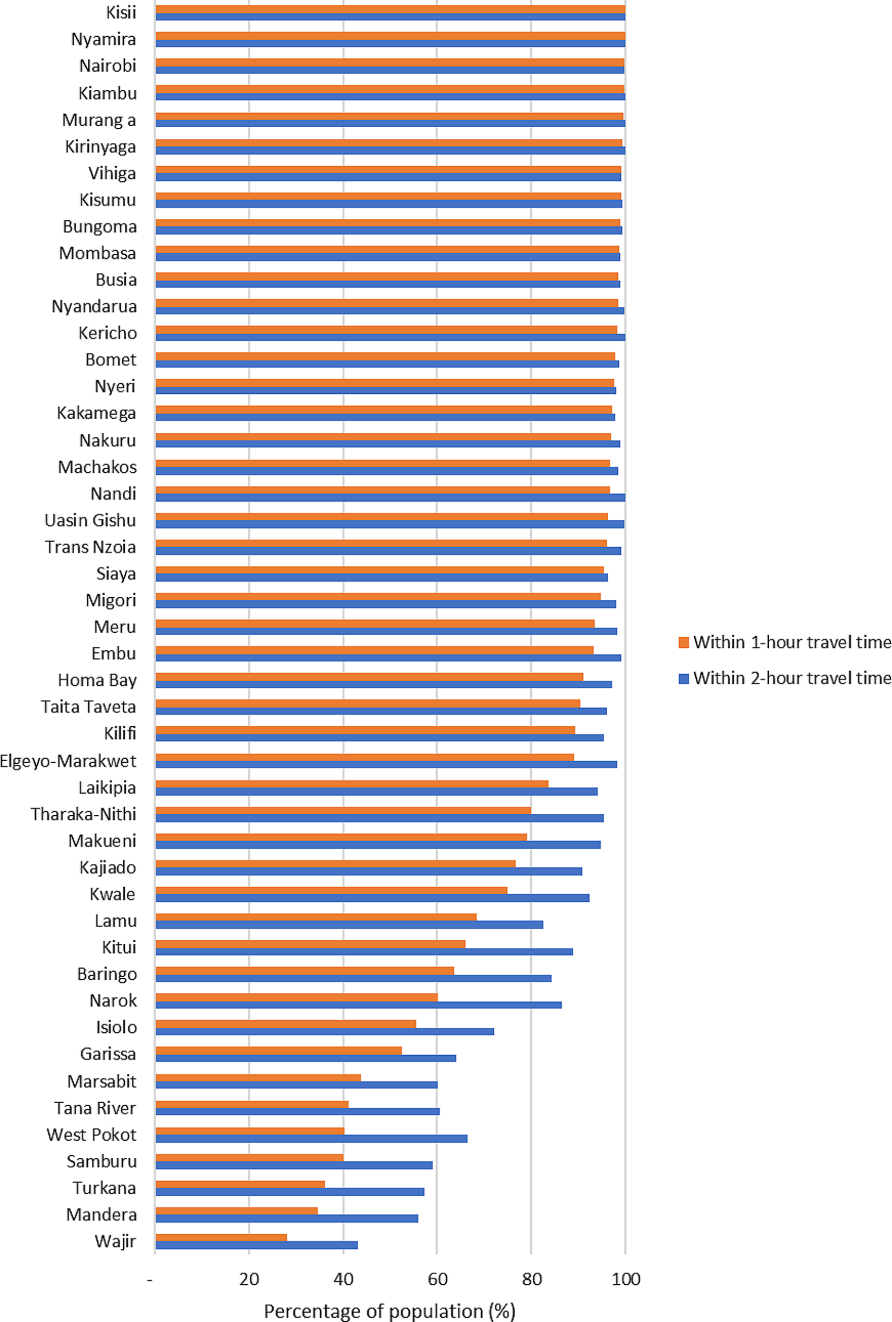
Distribution of the percentage of population living within 1- and 2-hour travel time to NHIF-contracted facilities

Nationally, 81.4% and 89.6% of the population lived within 60- and 120-minute travel time to an NHIF-contracted facility respectively (Fig. 2). However, there were notable inequalities in spatial access to NHIF-contracted facilities across counties. For instance, the proportion of the population living with 1-hour (60 min) travel time to an NHIF-contracted facility ranged from as low as 28.1% in Wajir county to 100% in Nyamira and Kisii counties (Fig. 3). The proportion of the population living within a 120-minute travel time to an NHIF-contracted facility ranged from 43.0% in Wajir County to 100% in Nyamira and Kisii counties (Fig. 3). This means that only four counties (Kiambu, Kisii, Nairobi and Nyamira) have met the target of having their population living within 1-hour (60 min) travel time to a facility.

When NHIF-contracted facilities under comprehensive contract, contracted to offer outpatient services and inpatient services were considered, the national proportion of the population living within 1-hour travel time was 81.4%, 81.4%, and 80.5% respectively. County-level distributions for spatial access to NHIF-contracted comprehensive, outpatient and inpatient facilities were similar to those presented in Fig. 3 hence we did not present graphs for these results.

**Fig. 4:**
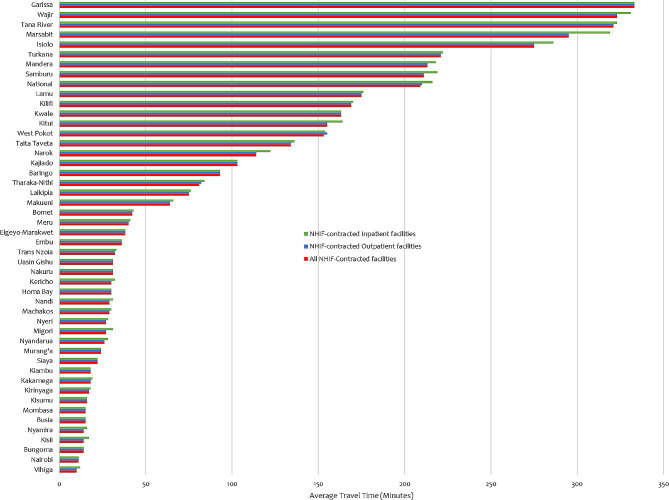
Average travel time to NHIF-contracted facilities nationally and at county level

On average, it takes 209, 210 and 216 min for a Kenyan to move from their household to any NHIF-contracted facility, an outpatient facility and inpatient facility respectively. There were wide inequalities in access to NHIF-contracted facilities at the county level. For instance, while the avarage travel time to any NHIF-contracted faciliy in Vihiga county was only 10-minutes, it was 33.3 times more for residents from Garissa County (Fig. 4).

## Discussion

Kenya has committed to accelerate progress towards UHC and has identified social health insurance as the strategy to drive the UHC agenda [25]. Consequently, understanding the spatial distribution of NHIF-contracted facilities in Kenya is crucial to informing health service access gaps, highlighting existing inequalities to access to facilities at the county level and examining progress to attaining national targets such as the government target to have 100% of the population living within 1 h travel time to a health facility (in this case an NHIF-contracted facility so that they can access the UHC cover). To the best of our knowledge, this provides the first evidence of spatial access to facilities contracted to a social health insurer in sub-Saharan African (SSA) countries even though most social health insurance schemes in SSA contract facilities to provide services on behalf of their beneficiaries [42, 43].

While findings at the national level highlight a high level of spatial access to NHIF-contracted facilities, this is still lower than the government target to have 100% of the population living within 1 h travel time (5 km) to a health facility [30]. Besides, we observed that spatial access to NHIF-contracted facilities is lowest, especially in marginalised counties. For instance, all counties (Narok, Isiolo, Garissa, Marsabit, Tana River, West Pokot, Samburu, Turkana, Mandera Wajir) that had less than 60% of their populations living within a 60-minute travel time to an NHIF-contracted facility are categorised as marginalised counties (based on access to clean water, road infrastructure, health and education indicators) in Kenya [44].

While the 2021 estimate that included all geocoded facilities in Kenya showed that 90% of the population lives within a 1-hour travel time to a health facility [19], our estimates indicate eight in 10 people to be within the required travel time to an NHIF-contracted facility. This has several implications. First, while overall spatial access estimates to health facilities provide crucial information for decision-making, not calculating specific spatial access estimates to contracted facilities may result in an overestimate of access. Second, while national-level estimates indicate higher proportions of the population living within the 1-hour travel time to an NHIF-contracted facility, we observed wide inequalities at the county level. This means that overall national spatial access estimates may mask existing inequalities at the sub-national level.

Findings from our study offer several policy implications. First, there is a need for the NHIF to fast-track the contracting of more health facilities across the country, especially in marginalised counties. This will still be the case as the NHIF transitions to a Social Health Insurance Fund (SHIF) following recent reforms [45]. This will enhance equity in access to care and remove availability barriers to accessing healthcare services [7]. Second, there is a need to redefine national spatial access targets and indicate NHIF-contracted facilities, especially as the country gears towards UHC. Calculating estimates based on all facilities may over-estimate actual spatial access hence mis-inform progress towards UHC.

### Study strengths and limitations

This study has several strengths and limitations. On strengths, first, this is the first study to examine spatial access to facilities contracted by a social health insurer in SSA and in Kenya both at the national and county levels. Second, the study utilised a harmonised database of geocoded facilities in Kenya. Third, like previous spatial access studies in Kenya, we accounted for travel barriers, travel speeds and modes of transport that influence access to health facilities.

On limitations, first, the list of facilities excluded prison and Kenya Defence Forces Memorial facilities and specialised facilities such as those for dental, diagnostics, and cancer specialised facilities. While these facilities were either not geocoded, not accessible to the general population and/or offered more specialised services, their exclusion may have underestimated the spatial access estimates presented in this paper. Second, the analysis did not account for the effect of seasonality and traffic jams due to data limitations. However, we are cognizant that these may influence travel speeds and modes of transport used (particularly in urban setting where for instancetraffic jams are rampant) hence affecting the overall spatial access estimates.

## Conclusion

Our study offers evidence of the spatial access estimates to NHIF-contracted facilities in Kenya that can inform contracting decisions at the NHIF or the recently formed SHIF, especially focussing on marginalised counties where more facilities need to be contracted. Besides, this evidence will be crucial as the country gears towards accelerating progress towards achieving UHC using social health insurance as the strategy to drive the UHC agenda in Kenya. Spatial access estimates from similar setting countries with public social health insurers that contract providers should consider estimating spatial access to contracted facilities as opposed to just all facilities to provide UHC-relevant evidence. Finally, while enhancing sptial access to contracted facilities is crucial, there is also a need to expand social health insurance coverage to the entire population to accelerate the country’s progress towards UHC.

## Data Availability

No datasets were generated or analysed during the current study.

## Abbreviations

FBO: Faith-Based Organizations
KEMRI: Kenya Medical Research Institute
KEPH: Kenya Essential Package for Health
KM: Kilometer
KMHFL: Kenya Master Health Facility List
MFL: Master Facility List
NHIF: National Health Insurance Fund
PHU: Population Health Unit
RCMRD: Regional Center for Mapping of Resources for Development
SDGs: Sustainable Development Goals
SHIF: Social Health Insurance Fund
SSA: sub-Saharan Africa
UHC: Universal Health Coverage
